# Inhibition of Alkbh5 Attenuates Lipopolysaccharide‐Induced Lung Injury by Promoting Ccl1 m6A and Treg Recruitment

**DOI:** 10.1111/cpr.70032

**Published:** 2025-04-20

**Authors:** Hongdou Ding, Xinnan Xu, Yaoyao Zhu, Xinyu Ling, Li Xu

**Affiliations:** ^1^ Department of Thoracic Surgery Shanghai Pulmonary Hospital, School of Medicine, Tongji University Shanghai China; ^2^ Department of Radiation Oncology Shanghai Pulmonary Hospital, Shanghai Pulmonary Hospital, School of Medicine, Tongji University Shanghai China

**Keywords:** ALI, Alkbh5, Ccl1, m6A, sepsis, Tregs

## Abstract

This paper discussed the role of AlkB homologue 5 (Alkbh5) in the progression of lipopolysaccharide (LPS)‐induced acute lung injury (ALI). LPS‐induced ALI models were established in Alkbh5 knockout (KO) and knock‐in (KI) mice. The m6A levels in lung tissues were analysed using m6A dot assays. The lung injury was analysed by determining ALI‐related markers and histological staining. Mouse MLE12 cells were exposed to LPS for in vitro experiments, and the influence of Alkbh5 on cell viability, apoptosis and reactive oxygen species (ROS) production was analysed. RNA‐seq analysis was performed to analyse gene changes upon Alkbh5 deficiency. Functions of the Alkbh5‐C‐C motif chemokine ligand 1 (Ccl1) cascade in ALI were further verified using the Alkbh5 antagonist DDO‐2728 and a recombinant protein of Ccl1 (mCcl1). Alkbh5 was upregulated in lung tissues following LPS exposure. Alkbh5 knockout in mice mitigated LPS‐induced lung injury, as indicated by reduced serum levels of lung injury markers and reduced immune cell infiltration, fibrosis and apoptosis. Conversely, Alkbh5 overexpression in mice resulted in reverse trends. In vitro, Alkbh5 knockdown in MLE12 cells enhanced cell viability while reducing cell apoptosis and ROS production. Mechanistically, Alkbh5 was found to bind to and destabilise Ccl1 mRNA, leading to increased Treg recruitment. Treatment with DDO‐2728 or mCcl1 in mice increased Treg infiltration, thus improving lung tissue pathology and reducing lung injury. This study suggests that Alkbh5 is implicated in ALI progression by reducing Ccl1‐mediated Treg recruitment, making it a promising target for ALI management.

## Graphical abstract

In septic lung injury mice, Alkbh5 is highly expressed, promoting the demethylation of m6A on Ccl1 mRNA, thereby reducing the stability of Ccl1 mRNA and decreasing Ccl1 expression. This, in turn, leads to reduced recruitment of Tregs, further exacerbating the symptoms of septic lung injury. The findings from this study suggest that Alkbh5‐mediated m6A demethylation plays a crucial role in modulating the inflammatory response in sepsis‐associated ALI by affecting Ccl1 mRNA stability and Treg dynamics. These insights contribute to a deeper understanding of the molecular mechanisms underlying sepsis‐induced ALI and highlight potential therapeutic strategies targeting m6A modifications to regulate immune responses in sepsis and other inflammatory diseases.

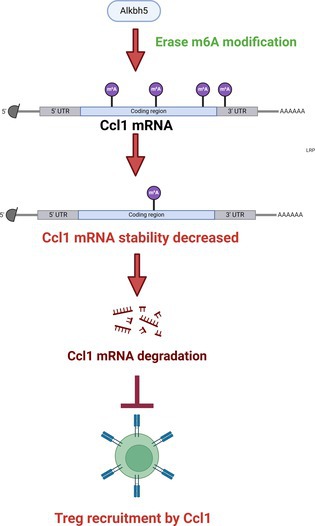

## 1 Introduction

Sepsis is a severe and often fatal condition characterised by an overwhelming systemic inflammatory response to infection, and it remains a leading cause of mortality in critically ill patients [1]. The lungs are particularly vulnerable to sepsis, often resulting in acute lung injury (ALI) and acute respiratory distress syndrome, which significantly contribute to the high mortality associated with this condition [2, 3].

Recent research has increasingly underlined the importance of epigenetic modifications, particularly N6‐methyladenosine (m6A), in regulating immune responses during inflammation [4, 5, 6]. m6A is the most prevalent internal modification on eukaryotic mRNA, with crucial roles in various aspects of RNA metabolism, including stability, splicing and translation [7]. The m6A modification is dynamically regulated by enzymes known as methyltransferases (‘writers’), demethylases (‘erasers’) and m6A‐binding proteins (‘readers’) [4]. AlkB homologue 5 (Alkbh5) is a key m6A demethylase involved in the modulation of mRNA stability and immune responses [8, 9]. Altered expression of Alkbh5 has been implicated in many pathological conditions [10]. In terms of inflammatory cascades, Alkbh5 loss has been associated with silica particles‐induced pulmonary inflammation [11]. However, reducing Alkbh5 levels has been demonstrated to reduce proliferation and inflammation in mouse glomerular mesangial cells [12]. Nevertheless, its role in sepsis and the associated organ injury remains largely unknown.

Chemokines and immune cells are pivotal in sepsis‐induced ALI [13, 14, 15, 16]. Among these, C‐C motif chemokine ligand 1 (Ccl1) is critical in orchestrating the immune response, particularly through the recruitment of regulatory T cells (Tregs) to sites of inflammation [17, 18, 19, 20]. Tregs, marked by the expression of transcription factor Foxp3, are essential for maintaining immune homeostasis and modulating excessive inflammatory responses [18, 21, 22]. In the context of sepsis, Tregs exhibit dual functions; they can attenuate the hyperinflammatory state, thereby protecting against tissue damage, or contribute to immune suppression, potentially exacerbating sepsis‐related complications [23, 24]. Beyond sepsis, Ccl1 and Tregs are implicated in diverse inflammatory diseases, like autoimmune disorders, chronic inflammatory conditions and cancer [25, 26]. Ccl1 can recruit Tregs to the tumour microenvironment, where they may facilitate immune evasion, and to chronic inflammation sites, contributing to sustained inflammation and tissue damage [18, 27]. In sepsis‐induced ALI, the interaction between Ccl1 and Tregs is particularly complex [28].

Given these insights, this study was conducted to analyse the functions of Alkbh5, particularly its role in m6A modification and Ccl1 mRNA modulation during ALI progression. The recruitment of Treg and the associated immune responses in this context will be explored as well.

## 2 Material and Methods

### 2.1 Animals and ALI Modelling

All animal procedures were approved by the Institutional Animal Care and Use Committee of Tongji University. Mice were maintained under specific pathogen‐free conditions with a 12‐h light/dark cycle and ad libitum access to food and water. To induce sepsis‐related ALI, mice were administered 5 mg/kg of lipopolysaccharide (LPS) (L2630, Sigma‐Aldrich) intraperitoneally. Lung tissues and serum were harvested 48 h post‐injection for subsequent analyses. Alkbh5 knockout (KO) and knock‐in (KI) mice were generated using adeno‐associated virus (AAV) vectors encaspulating short hairpin (sh) RNA or overexpression vectors of Alkbh5. KO mice were developed by introducing Alkbh5‐specific shRNA, while KI mice harboured a targeted expression construct leading to Alkbh5 overexpression. AAV9 viral vectors carrying the respective constructs were produced and purified using Addgene protocols, and were administered via intravenous injection at a dose of 1 × 10^11^ viral genomes (vg)/mouse. Wild‐type (WT) mice were administered control shRNA or plasmids. Quantitative polymerase chain reaction (qPCR) and western blot (WB) analyses were used to validate Alkbh5 expression levels in lung tissues. For pharmacological interventions, mice received intraperitoneal injections of the Alkbh5 antagonist DDO‐2728 (5 mg/kg, MCE) or recombinant mouse Ccl1 protein (mCcl1) (10 μg/kg, R&D system) 24 h prior to LPS injection. To investigate the role of Ccl1 in Treg recruitment, Alkbh5 KO mice were treated with the Ccl1/Ccr8‐specific antagonist R243 (0.3 mg/kg, Selleck) via intraperitoneal injection 24 h before LPS administration.

### 2.2 Cell Culture and Treatment

Murine MLE12 lung epithelial cells were obtained from ATCC and cultured in DMEM/F‐12 medium supplemented with 10% fetal bovine serum (FBS, Gibco), 1% penicillin–streptomycin (Gibco) and 1% L‐glutamine. Cells were maintained at 37°C in a humidified incubator with 5% CO_2_. For Alkbh5 knockdown, cells were transfected with Alkbh5‐specific shRNA or scrambled control shRNA using Lipofectamine 3000 (Thermo Fisher) according to the manufacturer's protocol. Knockdown efficiency was confirmed by qPCR and WB analysis 48 h post‐transfection.

### 2.3 LPS Treatment and Functional Assays

MLE12 cells were treated with LPS (1 μg/mL, Sigma‐Aldrich) for 24 h to mimic lung injury conditions. Cell viability was assessed using a cell counting kit‐8 (CCK‐8) kit (Dojindo), and apoptosis was evaluated by Annexin V/PI staining followed by flow cytometry. WB analysis was performed to detect apoptosis‐related markers, including Cleaved Caspase‐3, Bax and Bcl‐2.

### 2.4 Reactive Oxygen Species (ROS) Assessment

ROS levels were measured using the DCFDA (2′,7′‐dichlorofluorescein diacetate) assay. Briefly, MLE12 cells were incubated with 10 μM DCFDA (Abcam) at 37°C for 30 min, followed by fluorescence measurement using flow cytometry (BD LSRFortessa).

### 2.5 m6A Dot Blot Assays

Extraction of total RNA from lung tissues and cultured cells was performed using TRIzol reagent (15,596,026, Invitrogen). The m6A levels were assessed by dot blot analysis. In brief, 200 ng of total RNA was denatured at 65°C for 5 min, spotted onto a nitrocellulose membrane (Bio‐Rad, Cat. No. 1620115) and UV cross‐linked. The membrane was blocked in 5% non‐fat milk and incubated overnight at 4°C with anti‐m6A antibody (ab151230, 1:1000, Abcam), washed and incubated with HRP‐conjugated anti‐rabbit secondary antibody (ab205718, 1:5000, Abcam) for 1 h. The signal was detected using an enhanced chemiluminescence kit (Bio‐Rad, Cat. No. 1705060).

### 2.6 RNA Isolation and Quantification

Total RNA was isolated and reverse‐transcribed into cDNA using the High‐Capacity cDNA Reverse Transcription Kit (4,368,814, Applied Biosystems). Quantitative PCR was performed on an ABI 7500 Real‐Time PCR System using SYBR Green PCR Master Mix (4,309,155, Applied Biosystems). Relative expression levels were normalised to GAPDH and gauged with the 2^(−ΔΔCt) method. The following primers were used:

ALKBH5: Forward 5′‐TGGACTGGTTGCTGGAGAA‐3′, Reverse 5′‐CCAGCATCAGGCTGTTGTAG‐3′;

METTL3: Forward 5′‐GACTTCGTGGTGGTGGTGAT‐3′, Reverse 5′‐ATGCAGCTGGTGTGTTGAGA‐3′;

METTL14: Forward 5′‐CCAGTGGCTGGATGAGAACT‐3′, Reverse 5′‐GGTGACGACATCCACGTTCT‐3′;

FTO: Forward 5′‐ACGGTTACAGGCCGTAGATT‐3′, Reverse 5′‐CATTGACCATGCTTGTTGGA‐3′;

WTAP: Forward 5′‐GGAATGGCATGTTTGTGAAGC‐3′, Reverse 5′‐AAGCCATCTTGGCATCAAGAC‐3′;

Ccl1: Forward 5′‐TCTGCCTGTCCTGCTATTCA‐3′, Reverse 5′‐GCTGGTGGTAGATGGGTTGT‐3′.

### 2.7 WB Analysis

Lung tissues and MLE12 cells were lysed in RIPA buffer (Thermo Scientific, Cat. No. 89900) plus protease and phosphatase inhibitors (04693132001, Roche). Protein concentrations were quantified by the BCA kit (23227, Thermo Scientific). Proteins (30 μg per sample) were separated by 10% SDS‐PAGE and transferred onto PVDF membranes (IPVH00010, Millipore). Membranes were blocked with 5% non‐fat milk for 1 h and incubated overnight at 4°C with primary antibodies against ALKBH5 (ab195377, 1:1000, Abcam), METTL3 (ab195352, 1:1000, Abcam), METTL14 (ab220030, 1:1000, Abcam), FTO (ab92821, 1:1000, Abcam), WTAP (ab195380, 1:1000, Abcam), Ccl1 (ab9850, 1:1000, Abcam), Cleaved Caspase‐3 (9661, 1:1000, Cell Signaling Technology), Bax (2772, 1:1000, Cell Signaling Technology) and Bcl‐2 (2876, 1:1000, Cell Signaling Technology). After washing, membranes were probed with HRP‐conjugated anti‐rabbit IgG (7074, Cell Signalling Technology) and anti‐mouse IgG (7076, 1:2000, Cell Signaling Technology) for 1 h at ambient temperature. Protein bands were visualised using ECL reagent and imaged with the ChemiDoc MP Imaging System (Bio‐Rad).

### 2.8 Immunofluorescence and Fluorescence In Situ Hybridization (FISH)

Lung tissues were fixed in 4% paraformaldehyde, embedded in paraffin and sectioned at 5 μm thickness. Following deparaffinisation and rehydration, antigen retrieval was done in citrate buffer (pH 6.0). Sections were blocked in 5% BSA for 1 h and incubated overnight at 4°C with primary antibodies against ALKBH5 (ab195377, 1:200, Abcam), CK18 (ab668, 1:200, Abcam) and Foxp3 (ab20034, 1:200, Abcam). The next day, sections were probed with Alexa Fluor 488 or 594‐conjugated secondary antibodies (A‐11001 for 488, A‐11012 for 594, 1:500, Invitrogen) for 1 h. FISH was performed using a Cy3‐labelled Ccl1 mRNA probe (lnc110201, RiboBio). Slides were counterstained with DAPI (D1306 Invitrogen) and mounted using Fluoromount‐G (0100‐01SouthernBiotech). Images were captured using a confocal microscope (Leica SP8).

### 2.9 Enzyme‐Linked Immunosorbent Assays (ELISA)

Serum levels of KL‐6 (MBS702283, MyBioSource), SP‐D (SEA794Mu, Cloud‐Clone Corp), TNF‐alpha (MTA00B, R&D Systems) and CRP (ab99995, Abcam) were quantified using specific ELISA kits. Levels of Treg‐associated cytokines, TGF‐β1 (MB100B, R&D Systems) and IL‐4 (431,104, BioLegend) were measured. All assays were performed in triplicate, and absorbance was read at 450 nm on a microplate reader (EPOCH2, BioTek).

### 2.10 Histopathological Analysis

Lung tissue sections were stained with haematoxylin and eosin (HE), periodic acid‐Schiff (PAS), Masson's trichrome and terminal deoxynucleotidyl transferase (TdT)‐mediated dUTP nick end labeling (TUNEL) using standard procedures. HE staining was performed using haematoxylin (HHS32, Sigma‐Aldrich) and eosin (HT110216, Sigma‐Aldrich). PAS staining was conducted with the PAS Staining System (395B, Sigma‐Aldrich). MASSON'S trichrome staining was carried out using the Trichrome Stain Kit (Masson) (HT15, Sigma‐Aldrich). TUNEL was performed using the In Situ Cell Death Detection Kit (11684817910, Roche). Slides were examined under a light microscope (Nikon Eclipse Ci).

### 2.11 Immunohistochemistry (IHC)

Paraffin‐embedded lung tissue sections (4 μm) were deparaffinised, rehydrated and subjected to antigen retrieval using citrate buffer (pH 6.0) at 95°C for 20 min. Endogenous peroxidase activity was blocked with 3% hydrogen peroxide for 10 min, followed by blocking with 5% bovine serum albumin (BSA) for 1 h at room temperature. Sections were incubated overnight at 4°C with primary antibodies specific for Alkbh5, Foxp3 or other relevant markers. After washing, sections were incubated with HRP‐conjugated secondary antibodies for 1 h at room temperature, followed by DAB (3,3′‐diaminobenzidine) substrate development. Sections were counterstained with haematoxylin, dehydrated and mounted. IHC quantification was performed using ImageJ software. Five random fields per section were captured at 200× magnification, and the percentage of positive staining areas was calculated relative to the total tissue area. For cell counting, positively stained cells were quantified in at least three randomly selected high‐power fields (HPF, 400×).

### 2.12 MeRIP‐qPCR

Total RNA was extracted from lung tissues and cells. m6A‐modified RNA was enriched using the Magna MeRIP m6A Kit (17–10,499, Millipore). Briefly, 5 μg of total RNA was fragmented into ~100 nt fragments using RNA fragmentation reagent (AM8740, Ambion) and immunoprecipitated with anti‐m6A antibody (ab151230, Abcam) bound to magnetic beads. Following washing, the m6A‐enriched RNA was eluted and purified using phenol–chloroform extraction. cDNA was synthesised and qPCR was performed with primers specific for Ccl1 mRNA.

### 2.13 RNA Immunoprecipitation (RIP)‐qPCR

RIP was performed using the EZ‐Magna RIP Kit (17‐700, Millipore). Lung tissues or cells were lysed. Lysates were incubated with magnetic beads conjugated with anti‐ALKBH5 antibody (ab195377, Abcam) or anti‐FLAG antibody (F1804, Sigma‐Aldrich) overnight at 4°C. Immunoprecipitated RNA‐protein complexes were washed. RNA was extracted using phenol‐chloroform. The RNA was reverse‐transcribed into cDNA, and qPCR was performed to assess the enrichment of Ccl1 mRNA, with IgG as a negative control.

### 2.14 RNA Pull‐Down Assays

RNA pull‐down assay was designed to determine the direct interaction between Alkbh5 and Ccl1 mRNA. Biotin‐labeled RNA probes corresponding to Ccl1 mRNA (B01001, RiboBio) were transcribed in vitro using the MEGAscript T7 Kit (AM1334, Invitrogen). The probes were incubated with streptavidin magnetic beads (65602, Invitrogen) at 4°C for 1 h. Lung tissue lysates or MLE12 cell lysates were then incubated with RNA‐bound beads overnight at 4°C. After washing, the bound proteins were eluted and analysed by Western blotting using an anti‐ALKBH5 antibody (ab195377, Abcam).

### 2.15 Statistical Analysis

Data analyses were conducted utilising Prism 9 software (GraphPad, La Jolla, CA, USA), and results are expressed as the mean ± SD. Each experiment included at least three repetitions. Statistical comparisons were carried out using *t*‐tests or one‐way/two‐way ANOVA with Tukey's post hoc test, as suitable. A *p* value of less than 0.05 was considered statistically significant.

## 3 Results

### 3.1 m6A Modification and Alkbh5 in LPS‐Induced ALI

**FIGURE 1:**
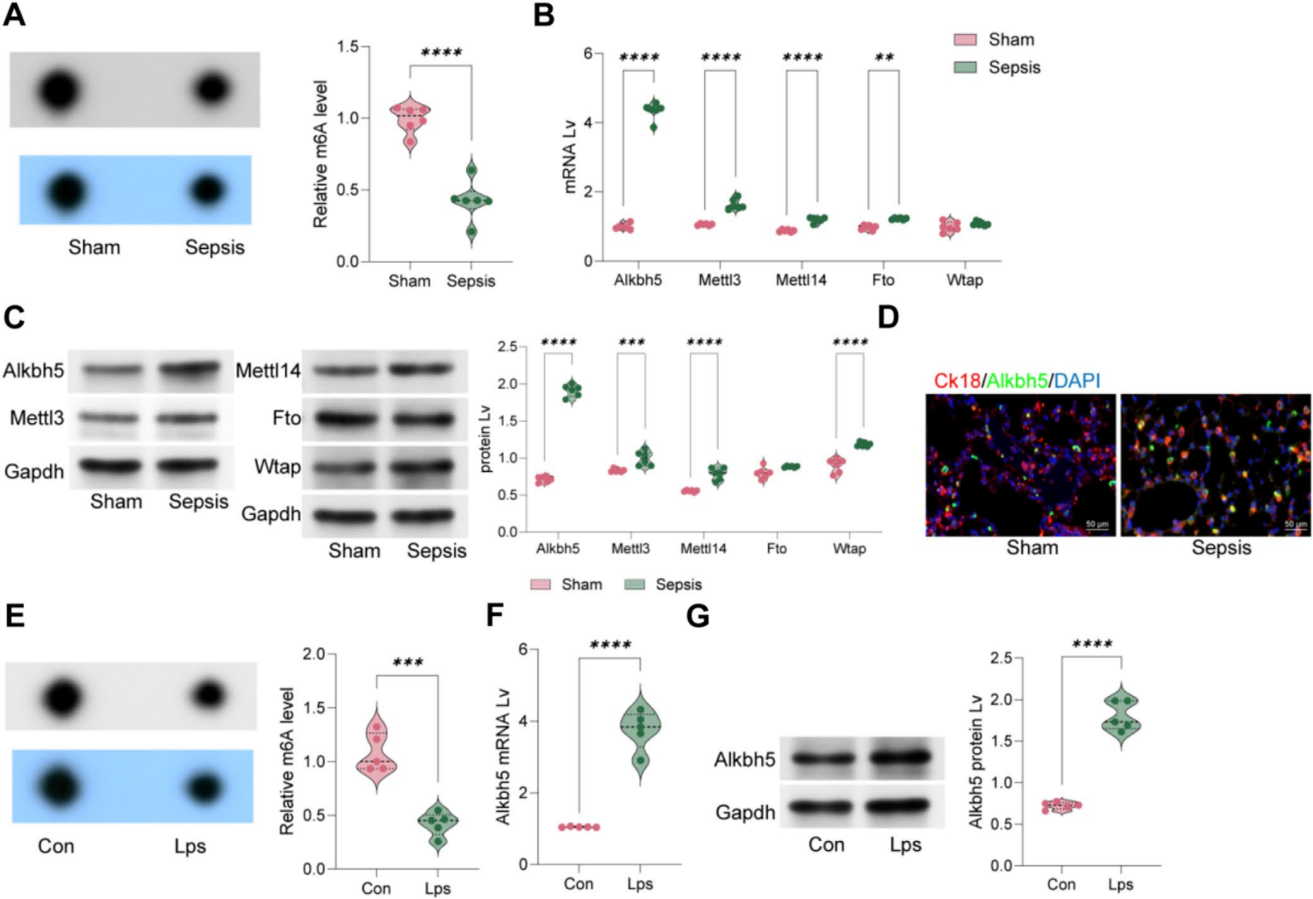
m6A modification and Alkbh5 involvement in LPS‐induced ALI. Sepsis‐associated ALI was induced in mice through LPS challenge. (A) m6A dot assays to assess the m6A levels in lung tissues of LPS‐induced mice; (B, C) qPCR and WB to detect the mRNA and protein levels of m6A modification‐related genes Alkbh5, METTL3, METTL14, FTO and WTAP in LPS‐treated lung tissues; (D) immunofluorescence to detect the expression levels and localization of CK18 and Alkbh5 in tissues; (E) m6A dot assays to assess m6A levels in LPS‐treated MLE12 cells; (F, G) qPCR and WB to detect the mRNA and protein levels of Alkbh5 in LPS‐treated cells. Each group contained six mice, and cell experiments were repeated at least three times. Data are presented as dot and violin plots and were statistically analysed using the unpaired *t* test (A, E–G) or two‐way ANOVA followed by Tukey's multiple comparison (B and C). **p < 0.01, ***p < 0.001, ****p < 0.0001.

m6A levels were notably diminished in lung tissues post‐LPS treatment (Figure 1A). Among several known m6A methyltransferase‐ and demethylase‐related proteins, Alkbh5 showed the most notable change, with its expression significantly increasing after LPS induction (Figure 1B,C). Subsequent fluorescence co‐localisation experiments demonstrated that Alkbh5 primarily localises in lung epithelial cells (Figure 1D). To validate these findings in vitro, we treated MLE12 cells with LPS and observed similarly decreased m6A levels and significantly increased Alkbh5 expression (Figure 1E–G).

### 3.2 Alkbh5 Modulation Influences LPS‐Induced ALI in Mice

**FIGURE 2:**
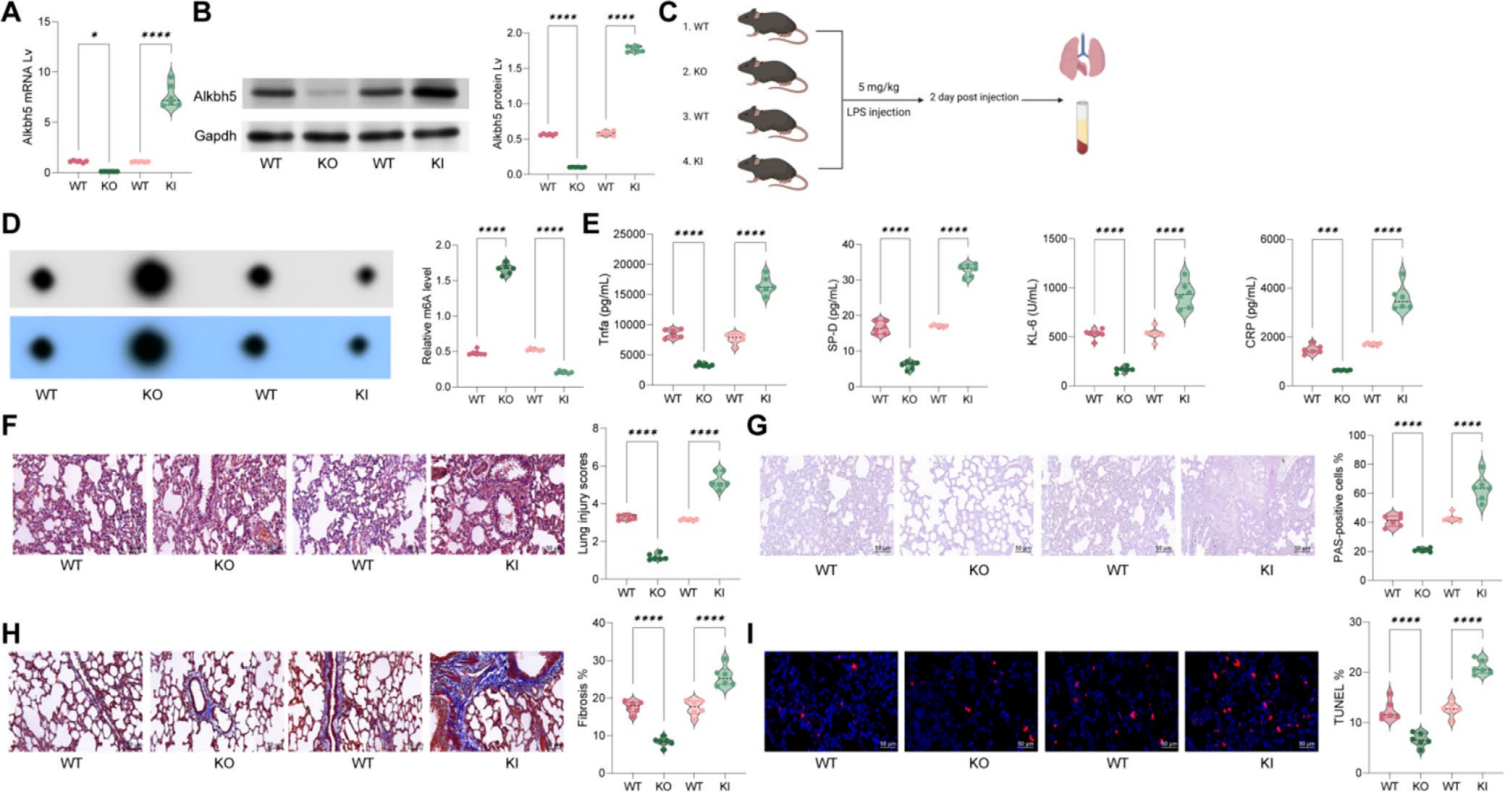
Knocking in or knocking out Alkbh5 alters LPS‐induced lung injury. Alkbh5 knockout (KO) and knock‐in (KI) mice were generated using AAB vectors‐encapsulating shRNA or overexpression vectors of Alkbh5. (A, B) qPCR and WB to detect the knockout and overexpression efficiency of Alkbh5 in lung tissue from KO and KI mice. (C) On Day 3, a sepsis‐induced lung injury model was established by intraperitoneal injection of LPS, and lung tissues and serum were collected 48 h later for further analysis; (D) m6A dot assays to assess m6A levels in lung tissues of LPS‐induced mice; E, ELISA to detect the levels of KL‐6, SP‐D, TNF‐alpha and CRP in mouse serum; (F–I) HE staining (F), PAS staining (G), Masson's trichrome staining (H) and TUNEL (I) assays to detect pathological changes, immune cell infiltration, fibrosis levels, and apoptosis in lung tissues of each group of mice, respectively. Each group contained six mice. Data are presented as dot and violin plots and were statistically analysed using one‐way ANOVA followed Tukey's multiple comparison (A, B, D–I). *p < 0.05, ****p < 0.0001.

qPCR and WB analyses confirmed the successful knockout and overexpression of Alkbh5 in lung tissues from KO and KI mice (Figure 2A,B). On Day 3, we established an LPS‐induced sepsis lung injury model by intraperitoneally injecting LPS and collected lung tissues and serum 48 h later for analysis (Figure 2C). m6A dot assay results showed that m6A levels in lung tissues were significantly elevated in KO mice but lowered in KI mice (Figure 2D). Moreover, serum levels of lung injury markers KL‐6, SP‐D, TNF‐alpha and CRP were greatly diminished in KO mice but increased in KI mice (Figure 2E). Histological analyses (HE, PAS, Masson's trichrome and TUNEL assays) revealed that KO mice exhibited a marked reduction in lung injury severity, immune cell infiltration, fibrosis and apoptosis, while KI mice showed exacerbated lung injury (Figure 2F–I).

### 3.3 Alkbh5 Knockdown Mitigates LPS‐Induced MLE12 Cell Injury

**FIGURE 3:**
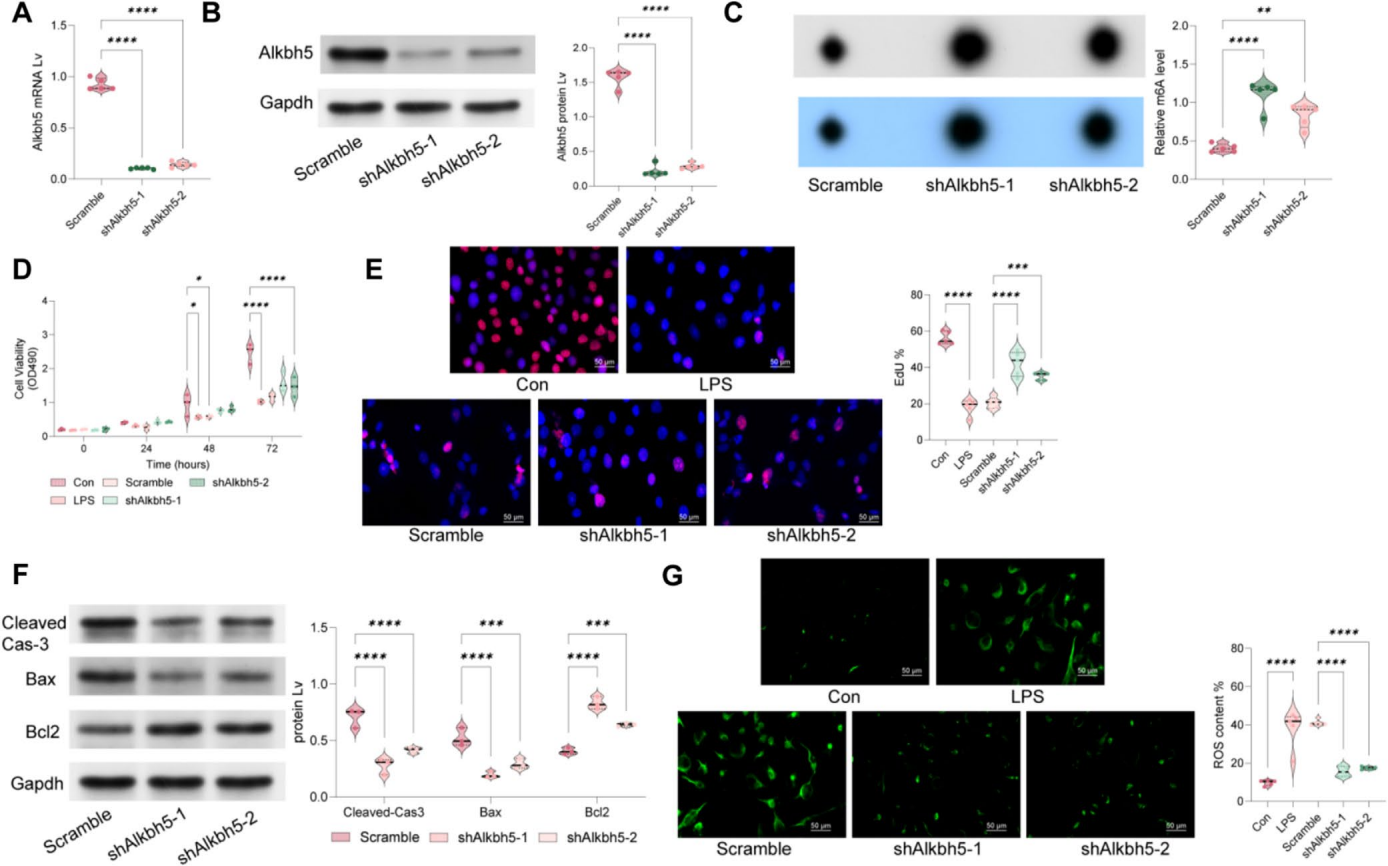
Alkbh5 knockdown inhibits LPS‐induced damage in MLE12 cells. Mouse MLE12 cells were transfected with shRNAs targeting Alkbh5 or a scramble shRNA. (A, B) qPCR and WB to detect changes in mRNA and protein levels of Alkbh5 in MLE12 cells. The cells were then treated with LPS. C, m6A dot assays to assess m6A levels in LPS‐induced MLE12 cells; (D, E) CCK‐8 and EdU to evaluate the activity of MLE12 cells; (F) WB to detect the protein levels of apoptosis‐related markers Cleaved Cas‐3, Bax and Bcl‐2 in MLE12 cells; (G) DCFDA staining to detect ROS levels in MLE12 cells. Cell experiments were repeated at least three times. Data are presented as dot and violin plots and were statistically analysed using one‐way (A–C, E and G) or two‐way (D and F) ANOVA followed by Tukey's multiple comparison. **p < 0.01, ***p < 0.001, ****p < 0.0001.

For in vitro experiments, we induced Alkbh5 knockdown in MLE12 cells (Figure 3A,B) and had the cells treated with LPS. Consistent with in vivo findings, Alkbh5 knockdown significantly increased m6A levels (Figure 3C) and enhanced cell viability (Figure 3D,E). Additionally, we analysed apoptosis‐related markers (Cleaved Cas‐3, Bax and Bcl‐2) and found that Alkbh5 silencing markedly decreased Cleaved Cas‐3 and Bax expression while increasing Bcl‐2 levels, thereby inhibiting LPS‐induced apoptosis (Figure 3F). Given that LPS induces ROS production, contributing to cellular damage, we used DCFDA staining and observed that Alkbh5 knockdown significantly reduced ROS production (Figure 3G).

### 3.4 Alkbh5 Directly Binds Ccl1 mRNA

**FIGURE 4:**
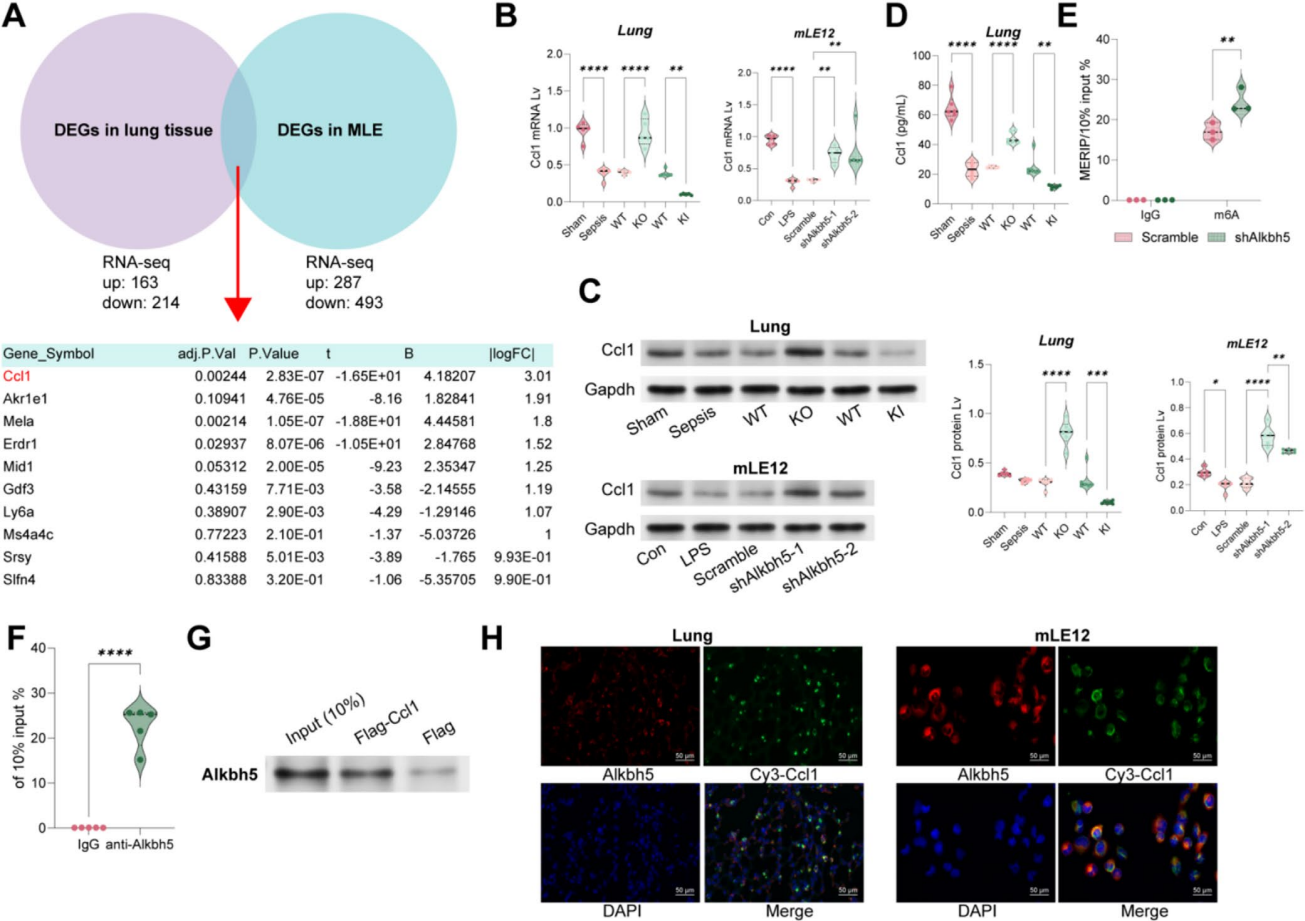
Alkbh5 directly binds to Ccl1 mRNA. A, RNA‐seq analysis to identify the top 10 differentially expressed genes in Alkbh5 KO mouse lung tissues and Alkbh5‐silenced MLE12 cells; (B, C) qPCR and WB to detect the mRNA and protein levels of Ccl1 in mouse lung tissues and MLE12 cells; (D) ELISA to detect the levels of Ccl1 in mouse serum; (E) m6A modification of Ccl1 mRNA was detected by MeRIP‐qPCR analysis using anti‐IgG and anti‐m6A antibodies; (F) relative enrichment of Ccl1 mRNA associated with Alkbh5 protein was identified by RIP assays using anti‐IgG and anti‐FLAG antibodies; (G) immunoblotting of Alkbh5 in IgG and Flag‐Ccl1 mRNA groups; (H) Cy3‐labelled Ccl1 mRNA FISH and anti‐Alkbh5 co‐localization in lung tissues and cells. Each group contained six mice, and cell experiments were repeated at least three times. Data are presented as dot and violin plots and were statistically analysed using one‐way (B–E) or two‐way (E) ANOVA followed by Tukey's multiple comparison, or analysed by the unpaired *t*‐test (E). *p < 0.05, **p < 0.01, ****p < 0.0001.

The RNA‐seq analysis showed that Ccl1 showed the most significant differential expression among the common genes altered in lung tissue of Alkbh5 KO mice and in MLE12 cells with Alkbh5 knockdown (Figure 4A). Further analyses demonstrated that the LPS induction significantly reduced Ccl1 mRNA levels, whereas Alkbh5 knockout or knockdown significantly increased Ccl1 mRNA expression (Figure 4B,C). ELISA results of Ccl1 expression in mouse serum were consistent with these findings (Figure 4D). MERIP‐qPCR experiments confirmed that anti‐m6A could pull down more Ccl1 mRNA, and Alkbh5 knockdown further increased Ccl1 mRNA enrichment (Figure 4E). Additionally, anti‐Alkbh5 could enrich Ccl1 mRNA, and reciprocally, Flag‐Ccl1 mRNA could pull down Alkbh5 (Figure 4F,G). FISH and immunofluorescence assays also confirmed that Alkbh5 binds to Cy3‐labelled Ccl1 mRNA (Figure 4H).

### 3.5 Alkbh5 Deficiency Enhances Ccl1 mRNA Stability Through m6A Modification

**FIGURE 5:**
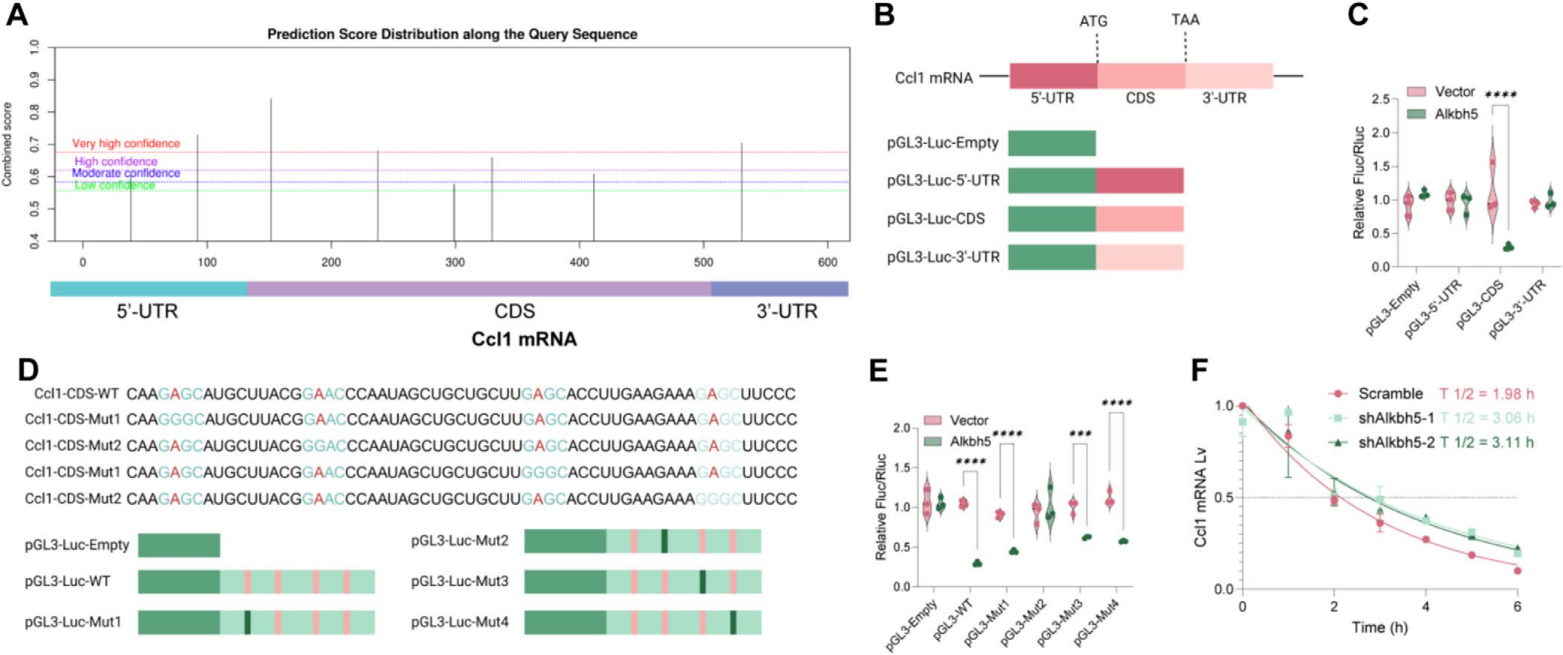
Alkbh5 deficiency enhances Ccl1 mRNA stability via an m6A‐dependent manner. (A) SRAMP website analysis of m6A modification sites on Ccl1 mRNA; (B) schematic diagram of pGL3 luciferase reporter vectors containing 5′‐UTR, CDS and 3′‐UTR sequences; (C) the Alkbh5 overexpression vector was co‐transfected with the constructed pGL3 luciferase reporter vector into 293T cells to analyse luciferase activity; (D) four mutated pGL3 luciferase reporter vectors were constructed; (E) the Alkbh5 overexpression vector was co‐transfected with the constructed pGL3 luciferase reporter vector into 293T cells to analyse luciferase activity; (F) qPCR to detect the half‐life of Ccl1 mRNA in cells. Cell experiments were repeated at least three times. Data are presented as dot and violin plots and were statistically analysed using two‐way ANOVA followed by Tukey's multiple comparison (C–F). ***p < 0.001, ****p < 0.0001.

m6A modification sites on Ccl1 mRNA were analysed using the SRAMP website (Figure 5A). Based on SRAMP analysis, m6A modification sites on Ccl1 mRNA are distributed across the 5′‐UTR, CDS and 3′‐UTR regions. pGL3 vectors containing these sequences were constructed (Figure 5B). The results elicited that Alkbh5 overexpression reduced the luciferase activity of pGL3‐CDS, but not pGL3‐5′UTR or pGL3‐3′UTR (Figure 5C,D). Further analysis revealed that Alkbh5 overexpression limited the luciferase activity in pGL3‐Ccl1‐WT and pGL3‐CCL28‐Mut1,3,4, but not in pGL3‐Ccl1‐Mut2 (Figure 5E). Given that Alkbh5 can influence mRNA stability via m6A modification [29, 30], we analysed the half‐life of Ccl1 mRNA in MLE12 cells. The half‐life of Ccl1 mRNA was 1.82 h in Alkbh5 knockdown cells, compared to 1.48 h in control cells (Figure 5F). Overall, Alkbh5 regulates Ccl1 mRNA stability through m6A modification.

### 3.6 Alkbh5 Deficiency Increases Ccl1‐Mediated Treg Recruitment

**FIGURE 6:**
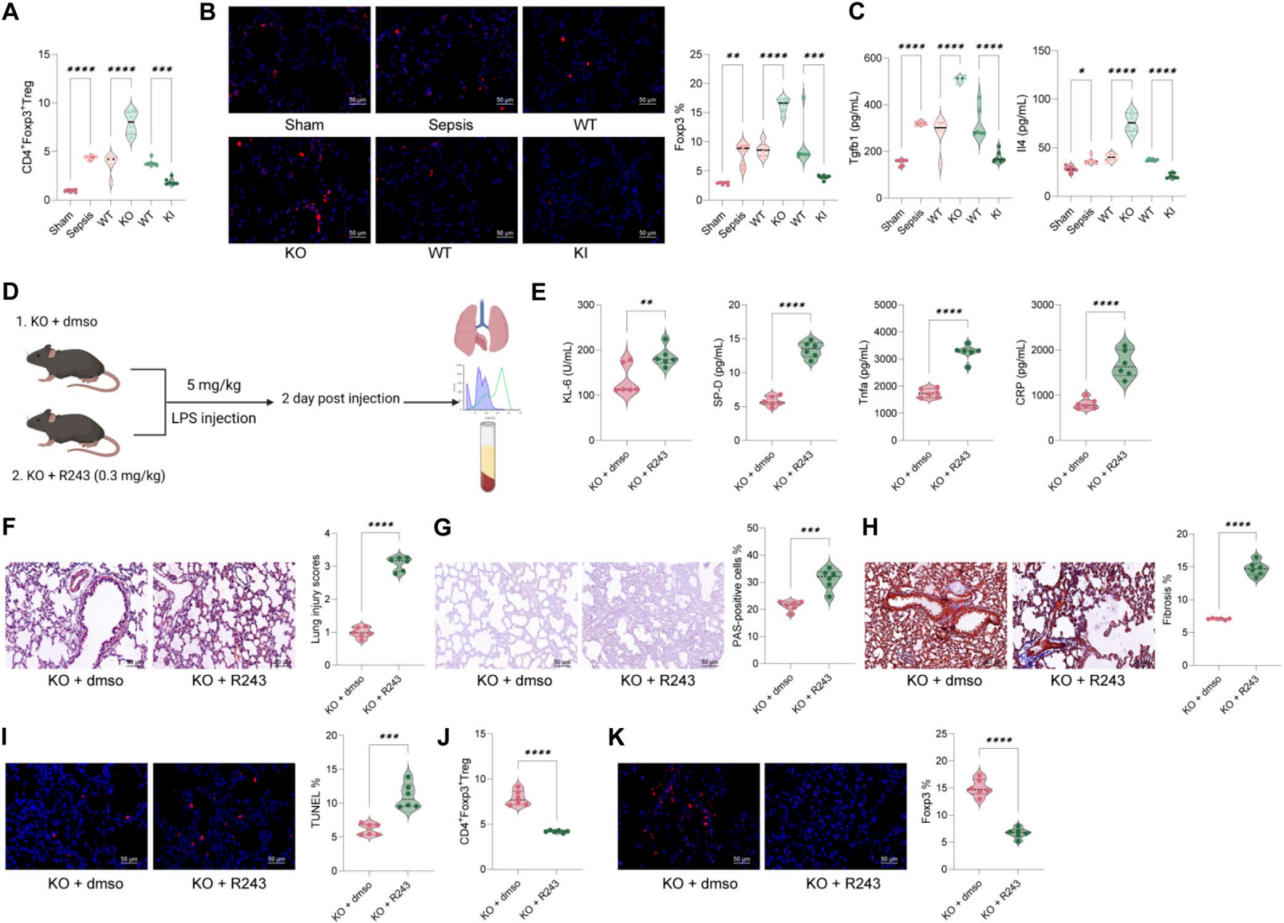
Alkbh5 deficiency increased the Ccl1‐mediated recruitment of Tregs. (A) Flow cytometry was used to detect the number of CD4+Foxp3+ Tregs in lung tissues; (B) Immunofluorescence to detect the number of Foxp3‐positive cells, a Treg marker, in mouse lung tissues; (C) ELISA to detect the levels of Treg cytokines Tgfb1 and Il4 in mouse serum; (D) Ccl1‐specific antagonist R243 (0.3 mg/kg) was used to treat KO mice; (E) ELISA to detect the levels of KL‐6, SP‐D, TNF‐alpha and CRP in mouse serum; (F–I) HE staining (F), PAS staining (G), Masson's trichrome staining (H) and TUNEL (I) assays to detect pathological changes, immune cell infiltration, fibrosis levels and apoptosis in lung tissues; (J) flow cytometry to detect the number of CD4+Foxp3+ Tregs in lung tissues; (K) immunofluorescence to detect the number of Foxp3‐positive cells, a Treg marker, in mouse lung tissues. Each group contained six mice. Data are presented as dot and violin plots and were statistically analysed using one‐way ANOVA followed by Tukey's multiple comparison (A–C), or analysed by the unpaired *t*‐test (E–K). *p < 0.05, **p < 0.01, ***p < 0.001, ****p < 0.0001.

Previous studies have shown that Ccl1 in lung tissue can recruit Tregs [31]. Tregs significantly increased in LPS‐induced lung tissue, likely due to the extensive immune cell infiltration during the initial immune response. In Alkbh5 KO mice, Treg numbers were further increased, while in KI mice, Tregs were significantly reduced (Figure 6A). Immunofluorescence detection of Treg marker Foxp3 in lung tissue yielded consistent results (Figure 6B). Additionally, serum levels of Treg cytokines Tgfb1 and Il4 were greatly enhanced in Alkbh5 KO mice but decreased in KI mice (Figure 6C). To confirm that Alkbh5 regulates LPS‐induced lung injury via Ccl1 stability, we treated KO mice with the Ccl1/Ccr8‐specific antagonist R243 (0.3 mg/kg). R243 treatment notably increased serum levels of KL‐6, SP‐D, TNF‐alpha and CRP (Figure 6E) and exacerbated lung injury and fibrosis (Figure 6F–I), while significantly reducing Treg infiltration (Figure 6J,K). These results indicate that Alkbh5 modulates Ccl1 mRNA stability, thereby affecting Treg infiltration and exacerbating LPS‐induced lung injury.

### 3.7 DDO‐2728 or mCcl1 Exerts Protective Effects on Sepsis‐Induced ALI

**FIGURE 7:**
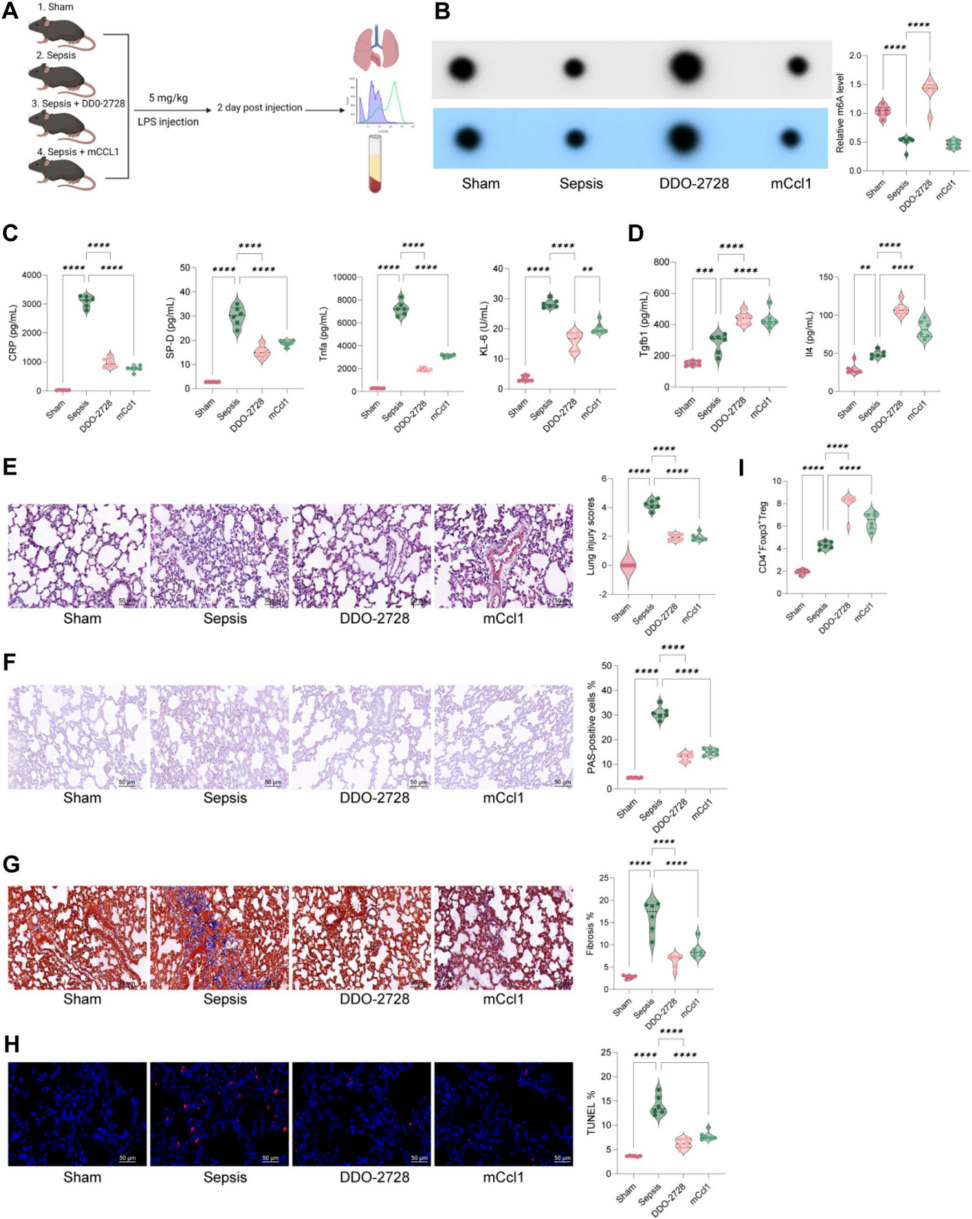
DDO‐2728 and mCcl1 exert protective effects in vivo during sepsis lung injury. (A) Commercial Alkbh5 antagonist DDO‐2728 and recombinant Ccl1 protein were used to treat LPS‐induced mice; (B) m6A dot assays to analyse m6A modification levels in mouse lung tissues; CD, ELISA to detect levels of KL‐6, SP‐D, TNF‐alpha and CRP, as well as Treg cytokines Tgfb1 and Il4 in mouse serum; (E–H) HE staining (F), PAS staining (G), Masson's trichrome staining (H) and TUNEL (I) assays to detect pathological changes, immune cell infiltration, fibrosis levels and apoptosis in lung tissues; (I) flow cytometry to detect the number of CD4+Foxp3+ Tregs in lung tissues. Each group contained six mice. Data are presented as dot and violin plots and were statistically analysed using one‐way ANOVA followed by Tukey's multiple comparison (B–F). **p < 0.01, ***p < 0.001, ****p < 0.0001.

Subsequently, we treated LPS‐induced mice with the Alkbh5 antagonist DDO‐2728 and recombinant Ccl1 protein (Figure 7A). DDO‐2728 treatment significantly increased m6A levels in lung tissue, while mCcl1 treatment did not show significant changes (Figure 7B). Treatment of either DDO‐2728 or mCcl1 significantly reduced serum levels of lung injury factors and increased Treg‐related cytokines (Figure 7C,D). Histological analysis revealed significant improvements in lung tissue pathology following DDO‐2728 and mCcl1 treatments (Figure 7E–H). Treg infiltration in lung tissue was also significantly increased after either treatment (Figure 7I). Overall, these results suggest that Alkbh5 exacerbates sepsis‐induced lung injury by destabilising Ccl1 mRNA via m6A demethylation, leading to reduced Treg infiltration. Conversely, targeting Alkbh5 or supplementing Ccl1 could offer therapeutic benefits in managing sepsis‐induced lung injury.

## 4 Discussion

RNA m6A modification modulators have been closely linked to the pathogenesis of inflammatory diseases including ALI [32, 33, 34]. This study suggests that Alkbh5 plays a significant pathological role in sepsis‐associated ALI by mediating m6A‐dependent Ccl1 mRNA degradation, which aggravates tissue damage and inflammation by reducing Treg recruitment.

Alkbh5 has been demonstrated to play diverse and complex roles in inflammatory conditions by influencing distinct downstream mRNAs. For instance, Alkbh5 loss has been linked to silica particles‐induced pulmonary inflammation [11]. Exposure to particulate matter has been found to reduce ALKBH5 protein levels in lung epithelial cells, leading to m6A‐dependent degradation of Atg13 mRNA and the activation of pro‐inflammatory pathways [35]. In contrast, LPS exposure has been demonstrated to increase Alkbh5 levels in mouse glomerular mesangial cells, triggering hyperproliferation and inflammation associated with chronic glomerulonephritis [12]. In a rat model of experimental colitis, administration of palmatine, a biologically active alkaloid, was found to prevent body weight loss and improve pathology by increasing m6A levels, which entailed the downregulation of Alkbh5 [36]. More relevantly, in a mouse model of cecal ligation and puncture‐induced polymicrobial sepsis, Alkbh5 was found to play a critical role in emergency granulopoiesis and neutrophil mobilisation to infection sites [37], key events that contribute to inflammation and tissue damage [38]. Partly consistent with previous studies, we observed that LPS challenge reduced m6A levels in both mouse lung tissues and MLE12 cells, with Alkbh5 identified as the most upregulated m6A demethylase. Regarding its role in ALI, we observed that Alkbh5 knockout in LPS‐challenged mice restored m6A levels, reduced serum levels of lung injury markers and mitigated immune cell infiltration, fibrosis and apoptosis. Conversely, Alkbh5 overexpression in mice resulted in reverse trends. In vitro, Alkbh5 knockdown in MLE12 cells enhanced cell viability while reducing cell apoptosis and ROS production. This evidence suggests the important pathological role of Alkbh5 in sepsis‐associated ALI.

In terms of the mRNA targets of Alkbh5 in the associated events, the RNA‐seq analysis identified Ccl1 as the most significantly differentially expressed mRNA in Alkbh5 KO mice and in MLE12 cells with Alkbh5 knockdown. Furthermore, we identified that Alkbh5 bound to the CDS of the mRNA, leading to RNA degradation through removing m6A modification. Ccl1 is well‐known to recruit Tregs to the inflammatory sites to inhibit overzealous inflammation [39]. Tregs can suppress the activity of pro‐inflammatory immune cells, such as effector T cells (e.g., Th1 and Th17 cells), which are often upregulated during sepsis, to reduce pro‐inflammatory signalling in the lungs [40]. Moreover, Tregs have been suggested to produce anti‐inflammatory cytokines, such as IL‐10 and TGF‐beta [41]. These cytokines not only help suppress inflammation but also promote tissue repair and regeneration [42, 43]. In sepsis‐associated ALI, these cytokines can limit lung injury and facilitate recovery. Indeed, increased differentiation and accumulation of Tregs have also been associated with inflammation resolution in mice with experimental sepsis‐associated ALI [44, 45]. In this study, we found thatAlkbh5 knockout in mice was found to increase Treg population, while Alkbh5 knock in resulted in reverse trends. Furthermore, we observed that treatment with the Alkbh5 antagonist DDO‐2728 or mCcl1 reduced serum levels of lung injury factors and increased Treg‐related cytokines, thus improving lung tissue pathology. This evidence verified that Alkbh5 mediates RNA degradation of Ccl1 to reduce Treg recruitment to the inflammation site, thus reducing inflammation resolution and aggravating sepsis‐associated ALI.

In summary, this study reports the critical pathological role of Alkbh5 in inflammatory damage in sepsis‐associated ALI. The RNA degradation of m6A and reduced recruitment of Tregs are, at least in part, entailed in these events. Targeting Alkbh5 or restoring Ccl1 levels to rescue inflammation resolution cascades holds the promise to control overzealous inflammatory damage in this context.

## Author Contributions

H.D. contributed significantly to the overall structure and content of the manuscript. X.X. was responsible for conducting the experimental work. X.L. served as the principal investigators of the study. L.X. assisted in the preparation of the manuscript, including editing and proofreading. All authors have reviewed the manuscript.

## Ethics Statement

Experimental procedures were ratified by the institutional animal care and use committee of Shanghai Pulmonary Hospital, School of Medicine, Tongji University (approval number: K24‐303Y). All animal housing and experiments followed the institutional guidelines for the care and use of laboratory animals.

## Conflicts of Interest

The authors declare no conflicts of interest.

## Data Availability

The data that support the findings of this study are available on request from the corresponding author. The data are not publicly available due to privacy or ethical restrictions.
